# Ageing or NOT, clock genes are important for memory processes: an interesting hypothesis raising many questions

**DOI:** 10.18632/aging.100148

**Published:** 2010-05-14

**Authors:** Oliver Rawashdeh, Jörg H. Stehle

**Affiliations:** ^1^ Department of Pharmacology and Toxicology, School of Medicine and Biomedical Sciences; University at Buffalo (SUNY), NY 14214 USA; ^2^ Institute of Cellular and Molecular Anatomy, Dr. Senckenbergische Anatomie, Goethe University Frankfurt; Germany

**Keywords:** Kondratova et al. Circadian clock proteins control adaptation to novel environment and memory formation. Aging. 2010: this issue

Memory processes, such as acquisition, consolidation and retrieval are temporally regulated events. An intrinsic circadian (*circa*: about; *dies*: day) timing system influencing the dynamics in memory processing has been detected in animal models, ranging from invertebrates to mammalian species. Several recent investigations, addressing the molecular mechanism behind the circadian modulation of mnemonic processes shed light onto pathways known to be essential in memory processing, such as the cAMP-CREB-MAPK-pathway, which by itself is regulated by the circadian system. Notable, an important role for rhythmic clock gene expressions in the hippocampus and in hippocampus-dependent memory processes has been recently detected.  However, the link between the circadian gating of memory processes and its importance for daily life remain highly elusive.

In the here presented work [1], Kondratova and co-workers approached the possible effects of ageing on cognitive performances, with the experimental design taking the known parallel decline in functional integrity of the circadian system and cognitive performance into account. For this purpose, the authors used 3-4 months old mice that are deficient for central clock genes, and thus, no longer exhibit circadian rhythms. Importantly, among the mice used, was the *Bmal1*-deficient mouse strain, known for its accelerated ageing phenotype. Kondratova and co-workers observed that behaviors, such as habituation to a novel environment were altered in mice with deficiencies/mutations of core clock genes. Habituation to a novel environment can be considered a form of non-associative learning, and is dependent on (a) hippocampus-dependent working memory, a form of short-term memory with regards to intra-session habituation and (b) long-term memory for inter-session habituation. *Bmal1*- and *Clock*-knockout mice showed deficiencies in both types of habituation in contrast to *Cry1/2*-deficient mice that demonstrated facilitated habituation. The authors also proved by using an open field test that elevated anxiety, which often correlates with high locomotor activity and rearing, and deficits in contextual habituation does not attribute to deficits in behavioral learning in the here used clock-gene*-*knockout mice: *Cry1,2*-knockout mice showed facilitated habituation in parallel to elevated levels of anxiety, while *Bmal1*-knockout mice showed deficits in contextual habituation at significantly lower anxiety levels.

The presented results confirm that a disruption of circadian clockwork impairs or facilitates in parallel the intra- and inter-session contextual habituation of mice. This observation acknowledges an essential regulatory role of core clock gene proteins in the herein analyzed behaviors, and thus in memory processes.

If this is the case, then the interesting hypothesis, that an age-associated dampening in the amplitude of rhythmic core clock protein expressions, or alternative "ageing associated imbalance between circadian clock proteins", can be linked to age-associated declines in mental performances.

Many questions remain: - Will an in depth comparative analyses of hippocampus-based behavior between wildtype and clock gene-deficient mice support the hypothesis? - Is the age-associated decline in clock gene expression hippocampus-specific, or a general phenomenon, and - Does the decline in clock gene expression amplitude or in phase-stability in the peripheral oscillator hippocampus, and/or in the brain circadian master clock residing in the hypothalamic suprachiasmatic nucleus (SCN) matter, with respect to the observed deterioration in mental performance? - Are the observed deficits in habituation in clock gene-deficient mice based on a hippocampal-intrinsic perturbation in time management, or - Is the disrupted temporal gating of hippocampal function related to the lesioned SCN clock in these knockout animals? - Is the observed impairment of long-term memory formation related to deficiencies of memory retrieval into working memory on the second day of habituation testing, rather than to impairments in memory consolidation? A clear answer to these urgent questions requires additional experiments, and demands the generation and investigation of a forebrain-specific conditional clock gene-knockout animal model. Then, the potential role of an ageing clockwork in the brain on mnemonic processes can be refined and attributed to a spatial component.

So while the time is ripe to acknowledge the effect of ageing on the time-of-day dependency of cognitive performances, much work remains to support the here hypothesized link between ageing associated decline in circadian behavior and memory formation.
